# Predicting Urban Heat‐Related Illness Across U.S. Climate Regions and Demographics

**DOI:** 10.1029/2025GH001655

**Published:** 2026-06-09

**Authors:** S. E. Brown, V. Shandas

**Affiliations:** ^1^ CAPA Strategies Portland OR USA; ^2^ Department of Geography Portland State University Portland OR USA; ^3^ Department of Geography Cramer Hall Portland OR USA

**Keywords:** heat‐related illness, CONUS, extreme heat event, MSA, simulation

## Abstract

We still know relatively little about how climate, demographics, built environment, and behaviors interact to drive hospitalizations during extreme heat events (EHEs). To address this paucity in understanding, we draw on extant literature to develop a transdisciplinary discrete event system dynamics (SD) modeling approach to address two research questions about the relationship between EHEs and heat‐related illnesses (HRIs): (a) How will changes in EHE frequencies, intensities, and durations in major Metropolitan Statistical Areas (MSAs) across the contiguous United States (CONUS) Climate Regions drive HRI morbidity across demographic, health‐status, and household groups over the coming decades? and (b) What are the anticipated HRI costs for these MSAs over the coming decades and how will the distribution of those costs evolve, across plausible low‐ and high‐emissions scenarios? By employing a system dynamics simulation model on a sample of the 53 largest population MSAs in CONUS, we produced stratified HRI and cost projections across regions out to the year 2040. The results suggest that differences across regions and scenarios depend on changing EHE profiles combined with underlying changes in the geographic distribution of demographic and socioeconomic disparities in risk. By combining continuous and discrete event modeling, this approach makes possible the construction of models which can be empirically tested at discrete points, both structural and parametric, along their causal chains. Such models may help align specific interventions that address community vulnerabilities to extreme heat while improving the calibration, coordination, and timing of regional responses.

## Plain Language Summary

Despite agreement on the need to better assess and mitigate heat‐related illness (HRI) risks, we still lack rigorous methods for modeling the complex interaction of risk factors over time. In this study we built a system dynamics model that simulates the occurrence of EHEs and HRIs in major MSAs across nine climate regions in the contiguous United States (CONUS) between 2005 and 2040. We first used previous studies to estimate the likelihood of getting sick from heat along a continuous range of heat index values depending on a person's age, general health, and household cooling behavior. We then incorporated climate projections of EHEs, demographic projections, and race‐based projections of household cooling behavior to estimate daily HRIs. We found that differences across regions and scenarios depend on changing EHE profiles combined with underlying changes in the geographic distribution of demographic and household cooling. By combining continuous and discrete event modeling, we are able to build and test models at discrete points along their causes‐and‐effects chains, even when those chains are complex. Such models can align specific interventions that address community vulnerabilities to extreme heat while improving the calibration, coordination, and timing of regional responses.

## Key Points

- We developed a system dynamics simulation model which we applied to a sample of the 53 largest MSAs in CONUS, by population
- We produced stratified heat‐related illness and cost projections across regions out to the year 2040
- The results indicate a reliable model for estimating heat‐related illnesses that are differentially experienced by region and demographics

## 1 Introduction

Despite growing concerns about the negative health impacts from extreme heat, we still know relatively little about how, and to what extent, increases in temperatures, particularly in cities where the majority of humans live and where the built environment amplifies temperatures, interact with co‐mediating factors such as demographics, health status, and cooling options to drive HRIs (Faurie et al., 2022; Hsu et al., 2021; Layton et al., 2020; McGeehan & Mirabelli, 2001; Sarofim et al., 2016; Vacellio et al., 2023; Voelkel et al., 2018; Wondmagegn et al., 2019). Studies have begun to estimate relative risks for HRIs as a function of heat index (HI), or similar measure, as mediated by region, pre‐existing condition, or other factors (Cheng et al., 2019; Metzger et al., 2010; Vaidyanathan et al., 2019; Wellenius et al., 2017; Ye et al., 2012). Such studies have improved our understanding of both same‐day and lagged HRI risks, especially at lower HI thresholds, leading in some cases to recalibration of local heat advisory thresholds. Further, studies show how past planning practices of race‐based segregation modified the built environment, creating surfaces and topographies that amplify temperatures and impact communities of color living in hotter neighborhoods (Gronlund, 2014; Hoffman et al., 2020). Multivariate models (both single and multi‐model) of the relationship between built‐environmental, demographic, and socioeconomic factors and HRIs have begun to quantify risks for especially vulnerable populations (Cardoza et al., 2020; Eisenman et al., 2016). Additionally, these studies suggest that exposure‐response functions are mediated by the degree to which a given extreme heat event (EHE) deviates from historical norms for the location under consideration, suggesting that acclimation is an important risk factor for HRIs (Wellenius et al., 2017), particularly when combined with mitigation actions (Georgescu et al., 2024).

However, even when historical studies of EHEs and HRIs consider changes over time, the disciplinarily limited scope of most earlier studies means that longitudinal models usually do not consider the interaction of diverse physical, demographic, and socio‐economic processes (Kinney et al., 2008). Recent studies have started to successfully build multidisciplinary models from historical data that, for example, project heat‐related mortality by region based on a combination of climate, demographic, and acclimation projections (Lee & Dessler, 2023). While these models improve on the quantification of HRI risk, they are typically constructed using inferential statistics (e.g., ordinary least square analysis, multivariate analysis, etc.) that rely on one or more time‐persistent (e.g., seasonal, decadal) variables, such as temperature averages and HRI incidence, measured over time (Choudhary & Vaidyanathan, 2014; Kingsley et al., 2016; Wang et al., 2016). While these statistical studies can reveal significant associations, they typically do not provide causal models that operationalize the interaction of factors during specific events occurring over time. There are still relatively few studies that use historical EHEs and their HRI outcomes, particularly at higher HIs where the exposure‐response relationship is likely to be less linear (Woods & Langer, 2023), to build and calibrate discrete event models and simulations that can be empirically tested and built upon to inform planning.

We aim to begin to fill this gap by developing and testing a discrete event simulation model that integrates currently disparate data sets and understandings about the implications of rising temperatures and associated heat related illnesses and costs in the contiguous United States (CONUS). System dynamics (SD) simulation modeling carries several advantages over other modeling approaches for such an undertaking. SD modeling uses a discipline‐neutral language of stocks and flows to describe accumulations and drainages over time (Morecroft, 2015; Sterman, 2000). Simulations run on the integration of ordinary differential equations based on delta time, or d(t), but SD simulation software packages can now model discrete events within the continuous simulation framework. The framework can therefore handle non‐linear relationships among continuous and discrete processes characterized by feedback loops and time delays. These systems may themselves be coupled into multi‐stock structures of varying complexity (Homer, 2014). This enables the analyst to elucidate structures and their parameters which explain observed system behavior. SD modeling can thereby account for complex and often counterintuitive behavior which econometric and similar modeling methods cannot (Sterman, 2008). And while disciplines often rely on modeling tools geared toward specific concepts, the SD modeling framework is flexible enough to integrate diverse disciplinary models and theories which facilitates the development and, importantly, operationalization of causal models that may be empirically tested and refined. Recently, SD modeling has been successfully applied to studies of ways to remediate risk from exposure to environmental hazards (Knappett et al., 2022), including extreme heat (Son et al., 2024).

The resulting transdisciplinary SD model projects trends in HRIs and costs out to 2040 by evaluating the potential implications of environmental, demographic, health status, and behavioral heat mitigation factors for likelihood of HRIs. This study, then, had four objectives: (a) to demonstrate how SD simulation can operationalize a causal model of HRI morbidity and costs that accounts for the interaction, over time, of diverse factors; (b) to begin to estimate at the national (CONUS) and regional (U.S. Climate Region) scales, HRI morbidity and costs over the coming decades, and as stratified by relevant demographic, health status, and household characteristics; (c) to identify, and begin to assess, both theoretical and empirical gaps in our models of HRIs and their uneven distributions; and (d) to thereby define a starting point for extending and refining the general model at more local scales that may inform planning to address different community vulnerabilities to extreme heat and HRIs. The SD model for this study, which covered the 53 largest MSAs in CONUS, by population, across the nine US Climate Regions and spanned between 2005 and 2040, was built on a general conceptualization of dynamically evolving population exposures to EHEs and downstream HRIs as mediated by demographic, health status, and household cooling factors.

## 2 Materials and Methods

### 2.1 Summary

The model was built on ordinary differential equations that used day as the time unit and differentiated each quarter day, that is, where d(t) = ¼ day. The National Environmental Modeling and Analysis Center Climate Explorer (nemac.org) projections of EHE‐days at defined daily T‐min and T‐max thresholds for MSAs under low‐ Representative Concentration Pathway (RCP 4.5) and high‐emissions (RCP 8.5) scenarios were used to sample discrete EHEs with varying frequencies, durations, and intensities, as measured using HI. For thresholds, we used T‐max greater than 100 degrees F (37.78°C) for the Northwest and T‐min greater than 80 degrees F (26.67°C) for all other regions. In this study, then, we treated EHEs as U.S. Climate Region‐wide events.

Population exposure for each region was estimated based on ongoing net population change including migrations of people in and out of the region. These net population flows were estimated from US regional population projections by the Weldon Cooper Center for Public Service, Demographics Research Group and stratified by age, health status, and household cooling status. Estimates of daily population exposures for each region were furthermore stratified by combining estimates of age, health status, and household cooling status derived from the Weldon Cooper Center for Public Service, Interactive Summary Health statistics for children and adults (National Health Interview Survey 2019–2022), and 2020 Residential Energy Consumption Survey (RECS), respectively.

Stratified daily all‐cause HRI flows were in turn estimated as products of these exposures and daily probabilities of developing an HRI as a complex function of these risk factors. Building on previous studies of proportional hazards across temperature ranges (Woods & Langer, 2023), a common logistic regression (sigmoidal) slope for the exposure‐response functions was derived by fitting to multiple constraints over an HI range from 100—130 degrees F (37.78–54.44°C). This included fitting stratified exposure‐response lines to data points from the one study of an EHE event in CONUS, the 2006 California HW, for which data on the EHE characteristics and resulting all‐cause HRI morbidity by strata were available (Knowlton et al., 2009). The logistic regression risk functions for the joint age, health status, and household cooling strata were furthermore assessed for (loose) face validity of relative risks across the HI range. Consistent with findings from previous studies on exposure‐response curves at HI thresholds below 105 degrees F (40.56°C) (Wellenius et al., 2017), each exposure‐response function was furthermore linearly extrapolated for HI values below 100 degrees F (37.78°C). A similar linear extrapolation above an HI of 130 degrees F (40.56°C) was made, although with no empirical basis since no studies yet provide data on exposure‐response functions above this threshold. Fractional (per exposed person) daily rates were simulated from these functions and combined with daily population exposures to EHEs to estimate HRIs, as well as costs, for relevant stratifications, including race, for each region and nationally.

### 2.2 Components of the Model

#### 2.2.1 Description of Scales Used

The 53 MSAs for this study were selected primarily to serve as a representative sample from the nine climate regions and do not reflect an exhaustive set (Table 1).

**Table 1:** Metropolitan Statistical Areas and Populations by U.S

US climate region	MSA	2022 population (est)
Northeast	New York‐Newark‐Jersey City, NY‐NJ‐PA	19,617,869
	Washington‐Arlington‐Alexandria, DC‐VA‐MD‐WV	6,373,756
	Philadelphia‐Camden‐Wilmington, PA‐NJ‐DE‐MD	6,241,164
	Boston‐Cambridge‐Newton, MA‐NH	4,900,550
	Baltimore‐Columbia‐Towson, MD	2,835,672
	Pittsburgh, PA	2,349,172
	Providence‐Warwick, RI‐MA	1,673,802
	Hartford‐East Hartford‐Middletown, CT	1,221,725
	Buffalo‐Cheektowaga, NY	1,161,192
	Rochester, NY	1,081,152
	Worcester, MA‐CT	980,137
Northern Rockies and Plains	Omaha‐Council Bluffs, NE‐IA	976,671
Northwest	Seattle‐Tacoma‐Bellevue, WA	4,034,248
	Portland‐Vancouver‐Hillsboro, OR‐WA	2,509,489
Ohio Valley	Chicago‐Naperville‐Elgin, IL‐IN‐WI	9,441,957
	St. Louis, MO‐IL	2,801,319
	Cincinnati, OH‐KY‐IN	2,265,051
	Kansas City, MO‐KS	2,209,494
	Columbus, OH	2,161,511
	Indianapolis‐Carmel‐Anderson, IN	2,141,779
	Cleveland‐Elyria, OH	2,063,132
	Nashville‐Davidson‐‐Murfreesboro‐‐Franklin, TN	2,046,828
	Memphis, TN‐MS‐AR	1,332,305
	Louisville/Jefferson County, KY‐IN	1,284,553
South	Dallas‐Fort Worth‐Arlington, TX	7,943,685
	Houston‐The Woodlands‐Sugar Land, TX	7,340,118
	San Antonio‐New Braunfels, TX	2,655,342
	Oklahoma City, OK	1,459,380
	New Orleans‐Metairie, LA	1,246,176
	Tulsa, OK	1,034,123
Southeast	Miami‐Fort Lauderdale‐Pompano Beach, FL	6,139,340
	Atlanta‐Sandy Springs‐Alpharetta, GA	6,222,106
	Orlando‐Kissimmee‐Sanford, FL	2,764,182
	Charlotte‐Concord‐Gastonia, NC‐SC	2,756,069
	Virginia Beach‐Norfolk‐Newport News, VA‐NC	1,806,840
	Jacksonville, FL	1,675,668
	Raleigh‐Cary, NC	1,484,338
	Birmingham‐Hoover, AL	1,116,857
Southwest	Phoenix‐Mesa‐Chandler, AZ	5,015,678
	Denver‐Aurora‐Lakewood, CO	2,985,871
	Salt Lake City, UT	1,266,191
	Tucson, AZ	1,057,597
Upper Midwest	Detroit‐Warren‐Dearborn, MI	4,345,761
	Minneapolis‐St. Paul‐Bloomington, MN‐WI	3,693,729
	Milwaukee‐Waukesha, WI	1,559,792
	Grand Rapids‐Kentwood, MI	1,094,198
West	Los Angeles‐Long Beach‐Anaheim, CA	12,872,322
	San Francisco‐Oakland‐Berkeley, CA	4,579,599
	Riverside‐San Bernardino‐Ontario, CA	4,667,558
	Sacramento‐Roseville‐Folsom, CA	2,416,702
	Las Vegas‐Henderson‐Paradise, NV	2,322,985
	San Jose‐Sunnyvale‐Santa Clara, CA	1,938,524
	Fresno, CA	1,015,190

*Note.* Climate Region.

The simulations used a daily time scale and ran on ordinary differential (flow) equations, differentiated each quarter day, from 2005 to 2040.

#### 2.2.2 Characterizing Extreme Heat Events

##### 2.2.2.1 Estimating Frequency of Extreme Heat Events

Frequency of EHEs depends in part on how one defines EHEs (Makido et al., 2019). For this study, except for the Northwest region, we used a daily minimum temperature (T min) threshold greater than 80 degrees F (26.67°C). This threshold was used to account for the extent to which the human body is or is not able to cool itself, particularly at night, during a heat wave. However, using this threshold predicted too few EHE days for the Northwest, where we instead used a daily maximum (*T* max) greater than 100 degrees F (37.78°C) threshold. The model used data provided through the NEMAC Climate Explorer Tool which provides Coupled Model Intercomparison Project Phase 5 (CMIP5) multi‐model projections of number of days in the year exceeding that threshold for major MSAs of each region. The NEMAC Climate Explorer projections for each MSA consisted of two sets of annualized time series of mean, minimum, and maximum (across all models) number of days per year exceeding the threshold: one set represented a low emissions (RCP 4.5) scenario, and one represented a high emissions (RCP 8.5) scenario. Time series for the major MSAs of each region were then averaged and used to interpolate daily mean number of EHEs for each region over the simulation time frame. These daily means were used to sample from a Poisson distribution to simulate the occurrence (or not) of EHEs on any given day, irrespective of EHE duration.

##### 2.2.2.2 Estimating Intensity of Extreme Heat Events

EHE intensity was separately estimated using HI which considers both ambient temperature and relative humidity and reflects how hot it feels (Smith et al., 2013). Heat index is relevant to HRIs because humidity can reduce the body's ability to cool itself. An HI was computed for each given EHE which lasted for the duration of the EHE. EHE duration was separately computed using a Beta distribution function with shape parameters α and β set equal to 2 and 5, respectively, corresponding to a probability density function with a positive (right) skew and bounded by a minimum and maximum. Importantly, EHE frequency, duration, and intensity were independently estimated and only indirectly related by their common use of a time‐dependent function.

Where appropriate, multi‐model studies were used to assist with parameterizing the sampling equations (Heutel et al., 2021). The model assumed that the natural log of the heat indices of EHEs follows a normal distribution with a specified mean and standard deviation. As a result, the log‐normal distribution of heat indices skews to the right. This assumption is appropriate for processes with large numbers of random inputs, but how well the log‐normal distribution fits to observed and projected HI data for CONUS for the period being studied was not evaluated here. Each time an EHE occurred, the model sampled from the log‐normal distribution to determine the HI of the EHE. The model used a starting mean intensity (set as an initial condition) and then linearly increased the mean intensity over the course of the 35 simulation‐years from 90 degrees F (32.22°C) (day 1) to 96.3 degrees F (35.72°C) (day 12,775). We arrived at the average decadal rate of increase in heat wave intensity from Zachariah et al. (2023) (Zachariah et al., 2023), whose study of the SW US/Mexico region found an average decadal increase in heat wave intensity of ∼1 degree C (1.8 degrees F). We applied this decadal rate of increase to all CONUS regions. Sensitivity analyses revealed that national cumulative HRIs through 2040 increased roughly 7‐fold over the generously large starting range of 80–100 degrees F (26.67–37.78°C), from 4.4 million to over 32.2 million. National cumulative HRI projections were also sensitive to variation in the standard deviation of EHE intensity, with a three‐fold increase in the standard deviation resulting in a 28.5‐fold increase, from 1.17 to 33.3 million, in HRIs over the course of the simulation.

##### 2.2.2.3 Estimating Duration of Extreme Heat Events

To calibrate the beta distribution function for EHE duration, the Beta distribution sample *X*, which ranges from 0 to 1, was rescaled to EHE duration (*Y*) in days using the transformation:

Y=a+X(b−a)

where, in this case, a and b are the minimum and maximum duration (in days), respectively, of the sample.

The model drew from Perkins‐Kirkpatrick and Lewis (2020), whose historical analyses of heat waves around the world showed that the Western U.S. region's annual maximum EHE duration (days) increased on average 0.2 days per decade between 1950 and 2017 (their study also showed an average of 1.17 additional heat wave days per decade for the Western U.S.). The 0.2‐day decadal increase in maximum annual EHE duration was the lowest rate increase of the major geographic areas examined in their study. Our simulation model therefore adjusted this rate upward by doubling to 0.4 days increase per decade. Sensitivity analysis indicated that model outputs were not particularly sensitive over a large range of assumed values for this parameter from 0.2–2 days. Over this range, for example, cumulative national HRIs increased from 11.2 to 12.4 million through 2040.

#### 2.2.3 Estimating Heat‐Related Illnesses

EHEs correspondingly “grow” and “diminish” stocks of people impacted as the EHEs occur and then dissipate. We characterized populations by three major individual risk factors for developing an HRI: age (0–4, 5–64,65+), overall health status (Excellent/Good, Fair/Poor), and the availability and use of household cooling (Uses AC, Uses Fans only, Neither). Population projections through 2040 for each region, by major age group, were sourced from the Weldon Cooper Center for Public Service, Demographics Research Group. Relative distribution of health status across populations was estimated as a general function of the age distribution using the Interactive Summary Health statistics for children and adults (C.D.C. (2026)):

%inpoorhealth=%veryyoung∗0.01+%youngandadult∗0.1+%olderadult∗0.25

Together, these two variables constituted the first layer of exposure. Household cooling status, measuring the extent to which the internal environment is controllable, defined a second layer of exposure. Data from the 2020 RECS were used and re‐coded to estimate, for each region, the availability and use of household cooling (Uses AC, Uses Fans only, Neither). For each region, the model multiplied the general prevalence of the three conditions, where health was itself conditioned on age, to arrive at a joint probability for each of the 18 strata:

jointprobabilityage,health,coolingstatus=p(age)∗p(healthstatus|age)∗p(householdcoolingstatus)

Daily HRI incidences were then computed for each region by multiplying the exposed population on any given day, stratified by age‐, health‐, and household cooling status, by their corresponding fractional (per person per day) rates for developing HRI. This rendered a daily HRI flow for each region and stratified by age‐, health‐, and internal climate control status, resulting in 3 × 23 × 9 = 162 distinct daily HRI incidence‐flows arrayed across the four dimensions:

HRIsperday=EHEpopulation∗p(age,health,AC)∗p(HRI|heatindex)/people/days

Fractional HRI rates (p(HRI HI)/people/days) were estimated from a logistic exposure‐response function of the HI of a given EHE and stratified by the three identified risk factors (age‐, health‐, and household cooling status). The function was estimated over an HI range between 100‐ and 130‐degrees F (37.78–54.44°C) and then (linearly) extrapolated at both ends of the range. Parameters of each function were estimated through stepwise checks and adjustments for reasonableness with respect to incremental HRI risk ratios, bounded by minimum and maximum HI values, across each of the three stratifications (Supporting Information S1). Here, we defined HRI as any heat‐related health condition resulting in an emergency department (ED) visit or hospitalization. The equations were further calibrated by comparing the model's simulated excess morbidity by age and race from the 2006 California heat wave (with known HI and duration) against Knowlton et al. (2009) estimates for the same event based on ED and hospital data. Comparison to the Knowlton et al. (2009) results however was complicated by differences in definitions of the race grouping, although comparisons of excess morbidity by age group could reasonably be made.

This simulation study assumed that the 18 corresponding functions do not vary across regions. Conditional probabilities of race affiliation, given the household's AC status and whether an HRI occurred in the past year, were also computed based on 2020 RECS data.

$$p\;(\text{Race}|\left(\text{AC}\;\text{status}\;⋂\;\text{HRI}\right)=\frac{p\left(\text{Race}\right)\;⋂\;p\left(\text{AC}\;\text{status}\right)\;⋂\;p\left(\text{HRI}\right)}{p\left(\text{AC}\;\text{status}\right)\;⋂\;p\left(\text{HRI}\right)}$$

National Census projections of future changes in race distribution were used to adjust from 2020 forward to 2040 (U.S. Census Bureau, 2023). Finally, an average healthcare cost of $5,359 (2023 US Dollars) per HRI was applied to arrive at estimates for total healthcare expenditures related to EHEs. This estimate comes from a Center for American Progress study (Woolf et al., 2023) of HRIs from 2016 to 2020 which looked at a mix of ED visits and hospital admissions, attributable to EHEs, across all diagnoses.

The 30‐run experiments consisted of Latin Hypercube sampling on the EHE frequency, intensity, and duration parameter space.

## 3 Data

NEMAC projections of major MSAs used to interpolate daily mean number of EHEs are available at Climate Explorer (nemac.org). National US population projections by race are available at Census Bureau Data (U.S. Census Bureau, 2023). US regional population projections by age are available at the Weldon Cooper Center for Public Service, Demographics Research Group Demographics | Cooper Center (Weldon Cooper Center for Public Service, 2024). Estimates for health status as a function of age were taken from the National Health Interview Survey 2019–2022, part of the Interactive Summary Health statistics for children and adults (C.D.C., 2026). The 2020 RECS data used to estimate distribution of household cooling statuses and to make race attributions of HRIs are available at Residential Energy Consumption Survey (RECS) ‐ Energy Information Administration (eia.gov) (U.S. Department of Energy, 2025).

## 4 Results and Discussion

### 4.1 Projections of National and Regional of Heat‐Related Illnesses Through 2040

**Figure 1:**
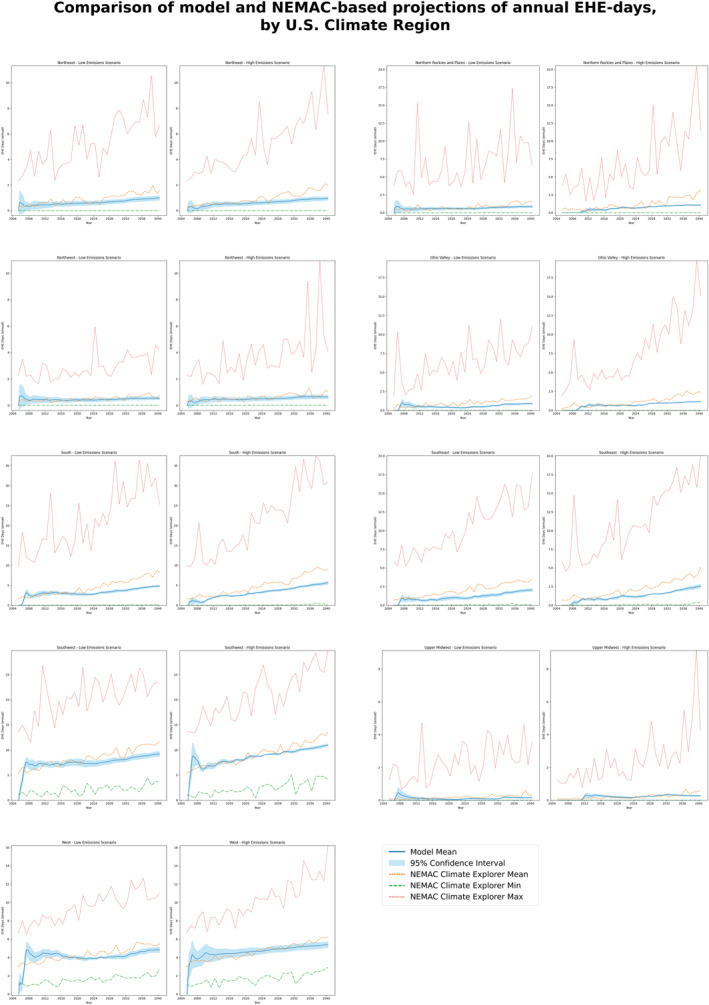
Comparison of model and NEMAC‐based projections of annual EHE‐days, by US Climate Region.

At the U.S. Climate Region level, the model generated 365‐day moving average annual EHE‐days that generally agreed with mean NEMAC projections under both low‐ and high‐emissions scenarios (Figure 1).

In terms of general population exposure, the projected annual EHE‐days for the 53 MSAs alone were estimated to impact approximately 26.1% of the CONUS population in 2012 (95% C.I. 10.1%–44.2%) and 33.5% of the CONUS population by 2040 (95% C.I. 23.8%–40.9%). Under the high emissions scenario, roughly 26.2% of the CONUS population were projected to be exposed to EHEs across these MSAs in 2012 (95% C.I. 8.8%–40.5%), increasing to 35% of the CONUS population by 2040 (95% C.I. 27.8%–43.9%).

The national average duration of EHEs, as defined in this study, was predicted by the model to be roughly 2.5 days (95% C.I. 0.9–4.1) in 2012 and increasing to 3.7 days (95% C.I. 2.9–4.5) in 2040. The national average intensity of EHEs, measured in terms of HI, was predicted to be roughly 97.6 degrees F (36.44°C) (95% C.I. 96.5–98.8) in 2012 and increasing to 99.4 degrees F (37.44°C) (95% C.I. 99.1–99.7) in 2040. EHE duration and intensity were estimated independent of frequency and were not sensitive to emissions assumptions. Projections of EHE duration and intensity relied instead on estimations from multidecadal historical studies (Perkins‐Kirkpatrick & Lewis, 2020; Zachariah et al., 2023; Zheng et al., 2021) to calibrate sampling parameters for (changing) maximum EHE duration and average EHE intensity.

Under the low emissions scenario, the model predicted 109,000 (95% C.I. 31.5k‐231k) HRIs nationally in 2012 and increasing to 217,000 by 2040 (95% C.I. 137k‐314k). Under the high emissions scenario, the model predicted 107,000 (95% C.I. 42.5k‐246k) HRIs nationally in 2012 and increasing to 237,000 by 2040 (95% C.I. 156k‐337k).

**Figure 2:**
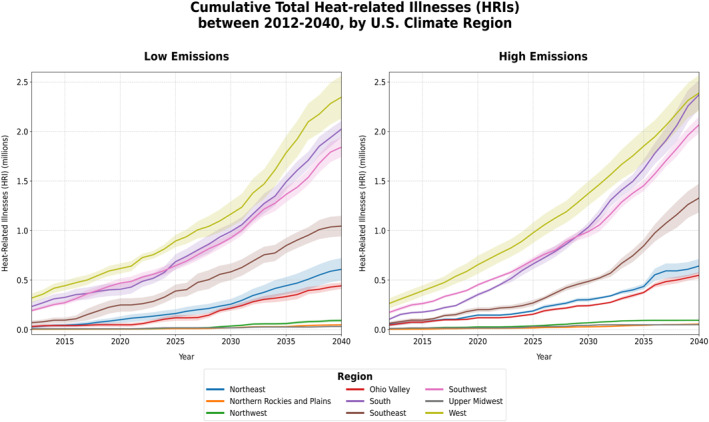
Running 95% Confidence Intervals of Total HRIs between 2012 and 2040, by US Climate Region (low‐ and high emissions).

The study measured HRIs in terms of ED visits or hospitalizations, regardless of diagnosis, resulting from exposure to extreme heat. The model predicted regional variation in HRI incidence, with the West climate region producing the highest incidence. Under multi‐run experiments (*n* = 30) that randomly varied draws from the same probability distributions for EHE frequency, duration, and intensity, regions showed somewhat distinct rates of growth in HRI frequencies, with some notable exceptions under the low emissions scenario, where HRI rates in the South and Southwest regions tracked one another closely while Northeast and Ohio Valley regions closely tracked one another before diverging in the late 2030s. The Northern Rockies/Plains and Upper Midwest regions however showed little to no difference in their HRI rates for either scenario. Figure 2 depicts the cumulative total number of HRIs projected by the model to occur for each region between 2012 and 2040 under the RCP 4.5 (low emissions) and 8.5 (high emissions) scenarios.

Examination of EHE, demographic, and projected household cooling trends in the model suggest that the relative acceleration of HRI incidence in the West, South, and Southwest, followed by the Southeast and Northeast, is largely driven by accelerating population growth combined with some aging in regions experiencing more EHEs, particularly in the Southeast, South, and West regions. Due to noise from model initialization, the first seven simulation years are not shown.

#### 4.1.1 Changing HRI Morbidities per Extreme Heat Event

**Figure 3:**
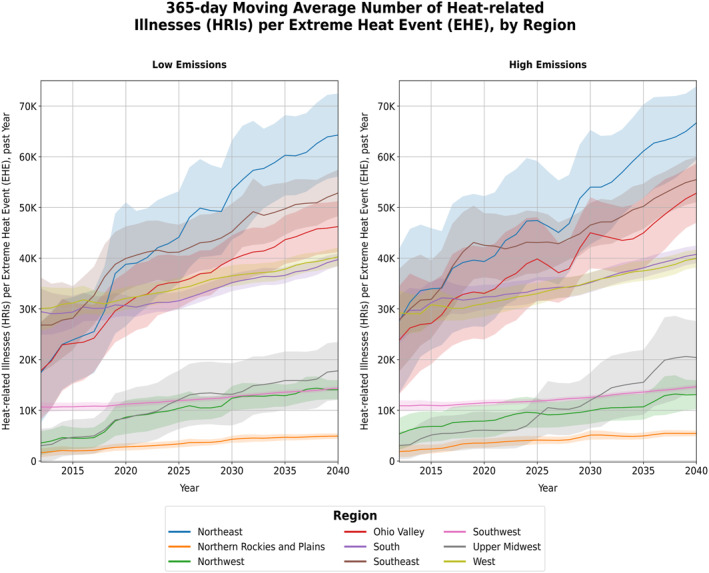
Plots of mean, with 95% confidence interval, number of HRIs per extreme heat event, by Region between 2012 and 2040 (low and high emissions).

The general model also allowed for a comparison of costs of EHEs, as measured by HRIs, both across regions and across time. Figure 3 depicts, for low‐ and high emissions scenarios, model estimations of the average number of HRIs per EHE between 2012 and 2040 for all nine U.S. Climate Regions.

The average HRI morbidity per EHE differs only slightly between the low‐ and high emissions scenarios through 2040. Within a given emissions scenario, however, the model predicted significant regional differences, not only in HRI incidence but in the evolution of that incidence.

The Northeast and Southeast regions, along with the Ohio Valley, were predicted to experience on average the largest number of HRIs per EHE over the coming decades, which is consistent with other studies (Heutel et al., 2021). Correspondingly, the model predicted similar regional variability in HRI costs as well as in the evolution of those costs, with the Northeast, Southeast, and Ohio Valley regions experiencing the costliest EHEs in terms of average number of HRI spending per event. However, morbidities and costs demonstrated some sensitivity to EHE sampling error, particularly for the Northeast, Ohio Valley, and Upper Midwest regions. For some simulation runs, the regions' annual EHE morbidity rankings for a given date range varied depending on the timing, duration, and intensity of EHEs. For regions with non‐overlapping 95% CIs, however, rankings were fairly stable across samplings. Generally, differences in the shape of regional trajectories were a consistent feature across samplings.

#### 4.1.2 Disparities in Projected Heat‐Related Illnesses

**Figure 4:**
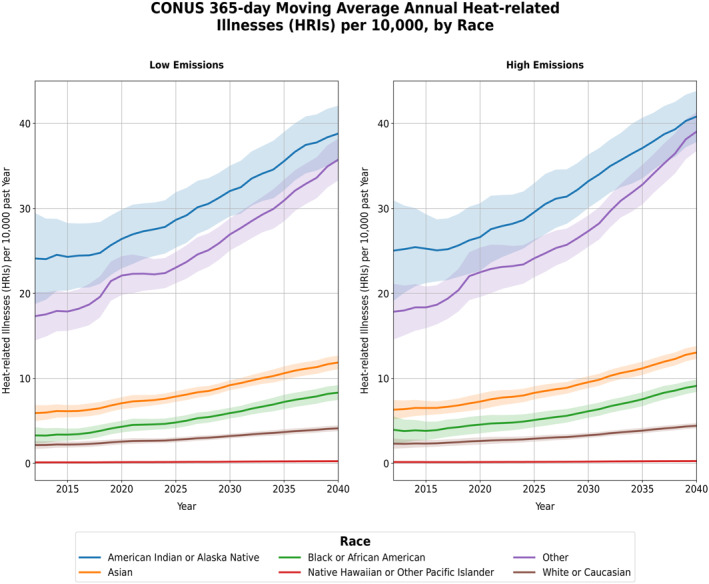
365‐day moving average, with 95% Confidence Interval, annual HRIs per 10,000, by Race (CONUS) (low and high emissions).

Using data from the 2020 RECS, conditional probabilities of race affiliation, conditioned on household cooling status and whether or not a person in the household experienced an HRI in the past year, were computed for each region. National Census projections by race through 2040 were then applied to arrive at national (for the CONUS MSAs sampled) HRI rates per 10,000 people for each major race grouping. Comparison of major race groups revealed significant differences not only in HRI incidence but also in changes in those rates over time (Figure 4). Aside from HRI incidence among Native Hawaiians/Other Pacific Islanders, which also showed high relative sampling error at 15.1%, estimated White HRI incidence was not only the lowest but showed the smallest increase over the course of the simulation timeframe.

Comparison of excessive morbidity predicted by the model to Knowlton et al. (2009) study of the 2006 California HW suggests that the model might be systematically underestimating HRI risk in the 65+ age group (Table 2). It should be noted however that the model's definition of the West region was comprised primarily of major California MSAs as well as Clark County, Nevada (Table 1), whereas the Knowlton et al. (2009) study looked at ED and hospital data for all of California.

**Table 2:** Comparison of Simulated and Estimated Excess ED Visits and Hospitalizations (All Diagnoses) Attributable to the 2006 California Heat Wave (July 15–1 August 2006), by Age Cohort

Age	Simulated excess ED visits and hospitalizations	Estimated excess ED visits and hospitalizations (Knowlton et al., 2009)
0–4	2,090	2,581
5–64	8,130	11,750
65+	1,600	3,017

**Figure 5:**
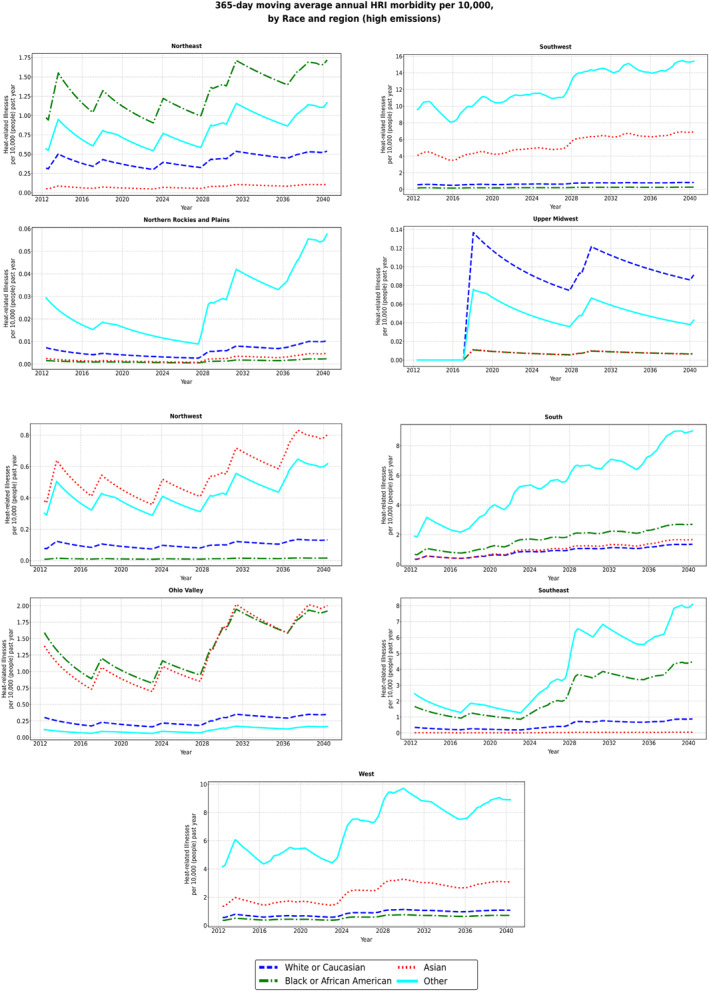
High emissions morbidity per 10,000 lives by four major race groups, African American/Black, Asian, Other (i.e., two or more race categories), and White, for each of the nine U.S. Climate Regions.

Figure 5 displays morbidity per 10,000 lives under the high emissions scenario by the four major race groups, African American/Black, Asian, Other (i.e., two or more race categories), and White) for each of the nine U.S. Climate Regions' major MSAs using default model sampling seeds. For comparison, scales vary.

We found important regional differences with respect to HRI rates within the same race categories. The model also allowed for comparisons to be made within regions that thereby controlled for regional differences in these EHE and HRI risk characteristics. We found regional differences in racial disparities in HRIs, not only at any given time, but also with respect to how those disparities evolved.

In terms of magnitude of disparities across the four race groups, the Northeast, Ohio Valley, South, Southwest, and to a lesser extent West, regions exhibited the largest disparities, with the highest risk group experiencing HRIs at a rate between roughly 12 (Ohio Valley) to over 57 (Southwest) times that of the lowest risk group. While these regional differences are notable, White populations, apart from the Upper Midwest, had consistently among the lowest HRI rates across regions and over time.

#### 4.1.3 National Heat‐Related Illness Cost Projections

**Figure 6:**
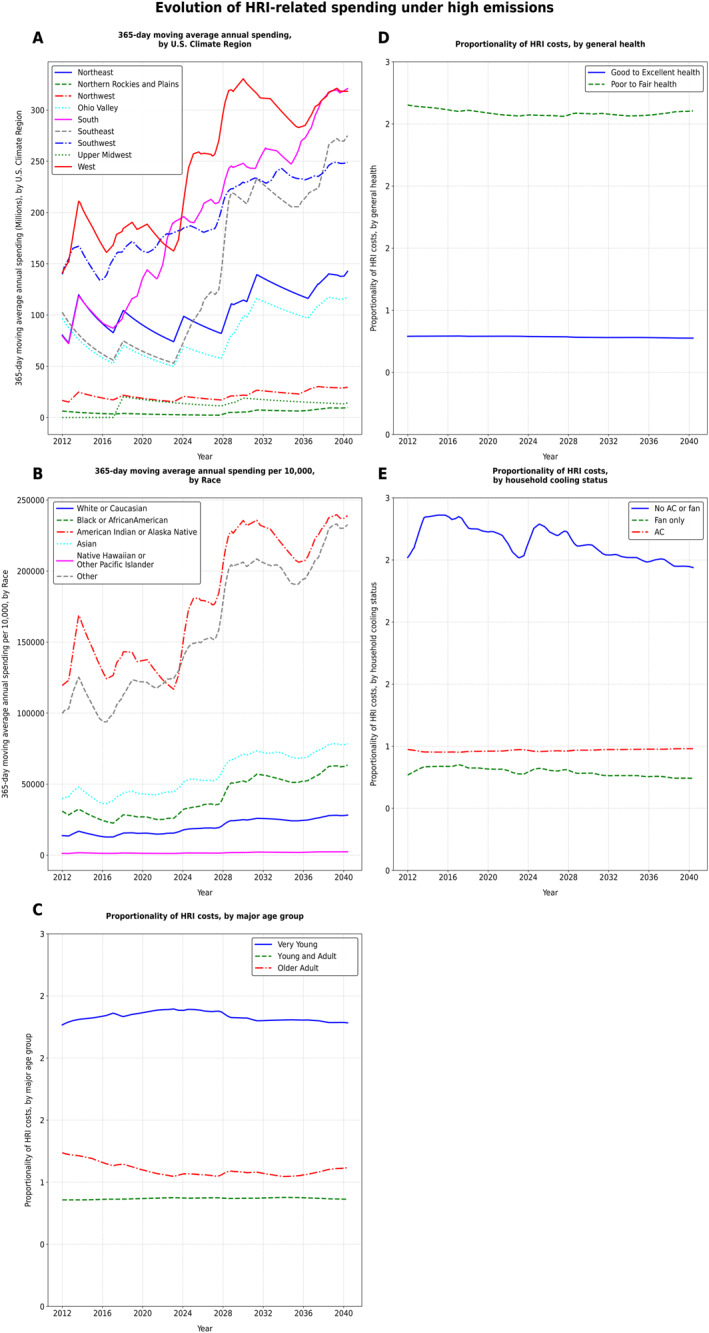
Evolution of HRI‐related spending under high emissions: 365‐day moving average annual spending by region (a); 365‐day moving average annual spending per 10,000, by Race (b); proportionality of heat‐related illness costs by major age group (c), by general health (d), and by household cooling status (e).

For the MSAs that were considered (Table 1), the model projected total HRI medical expenditures, measured in 2023 U.S. Dollars, in 2012 to be $582 million (95% C.I.$169 million‐1.24 billion) and $1.16 billion (95% C.I. $733 million‐1.68 billion) by 2040 under the low emissions scenario. Under the high emissions scenario, the model estimated total HRI expenditures to be $576 million (95% C.I. 228–1.3 billion) already in 2012, reaching $1.3 billion (95% C.I. $837 million‐1.8 billion) by 2040. Using the model default sampling seeds for EHE frequency, intensity, and duration, we modeled the changing distribution of these costs under the high emissions scenarios (Figure 6).

### 4.2 Future Regional EHEs and HRIs by Demographics

The model's projections for annual number of EHE‐days were within the bounds of other estimates (Larsen et al., 2017), although the precise definition of EHEs varies from study to study. The results, consistent with other studies, suggest that HRIs and their costs will not be evenly distributed across regions. The West region, represented by the CA MSAs of Fresno County, Los Angeles County, Riverside County, Sacramento County, San Francisco County, Santa Clara County, as well as Clark County, NV, is projected to experience the greatest number of HRIs, while the Northeast, Southeast, and Ohio Valley MSAs are expected to experience the costliest EHEs in terms of HRIs, which is consistent with other studies (McGeehan & Mirabelli, 2001). Data from the 2020 RECS suggests that the highest percentages of populations using no AC or fan are in the Northwest and West regions (7.2% and 5.9% respectively). EHE frequencies, intensities, and duration for these two regions therefore appear, at least in the near‐term, to be especially important drivers of national costs associated with HRIs. Whether this continues to hold true will depend in part on whether and to what extent the geographic distribution of ACs and related household cooling behaviors change.

Nationally, when comparing HRI morbidity by race, the model predicted the very young and American Indian and Alaska Native groups to be the most susceptible to HRIs. However, RECS survey RSEs for the estimated number of households self‐reporting experiencing any HRI in the past year were relatively large for most of the race categories. The group with the second highest annual HRI morbidity rate (per 10,000), Other, had better representation in the RECS data and provides a more meaningful assessment of racial disparities. The Other group consistently showed rates three times that of Asians, four times that of Blacks/African Americans, and between 9 and 10 times the rate of Whites over the course of the simulation. These findings suggest that race will continue to be an important risk factor for HRIs in the future.

Further comparisons at the national level of HRI risk by health and household cooling status showed similarly striking disparities. Over the course of a simulation, the changing geographic distribution of these risk characteristics, combined with changing frequencies, durations, and intensities of EHEs, produced sometimes counterintuitive results. Sampling only on EHE characteristics, we observed for example, that the national proportion of HRIs borne by AC households relative to their prevalence surpassed the proportion of HRIs among fan‐only households relative to their prevalence, which may be due in part to increasing frequencies of EHEs in regions with a higher prevalence of AC households. Similarly, the disproportionality of spending from HRIs in the 65 years and older age group decreased for a period of time before increasing again which may be due to an overall aging population combined with a growing relative concentration of EHEs in regions with older populations, especially in the Southeast and Southwest (Figure 6). These results are furthermore consistent with studies which suggest that the relationship between HRI risk and home cooling technologies is often mediated by other factors including energy costs (Eisenman et al., 2016). National results showed sensitivity to sampling error in EHE frequencies, duration, and intensities, although rankings across groups were relatively stable across samplings.

The model also suggests that, when regional differences in the noted EHE and HRI risk factors are controlled for, there remain significant disparities in HRI risks across race groups. Furthermore, geography, health status, and cooling behaviors are all likely interacting to contribute to the variation in race disparities. The complexity of these interactions is moreover enhanced to the extent that they are mediated by institutional processes. The findings suggest significant opportunities for mitigating racial HRI disparities in these regions. In addition to overall disparities, regions also vary in the ranking of HRI morbidities across groups, suggesting that interventions to reduce disparities will need to be place‐based, meaning they take into consideration the history of heat events, local geographies, population sensitivities, and the distribution of mitigation and communication resources. Finally, baseline projections suggest that different trajectories across regions are likely, with some regions seeing disparities increasing and even accelerating over time (Figure 5).

#### 4.2.1 Limitations and Opportunities for Improving Models of EHEs and HRIs

A third objective of the study was to identify, and begin to assess, both theoretical and empirical gaps in our models of HRIs and their uneven distributions. Our aim was to build a sufficiently operationalized discrete event simulation model that could begin to elucidate how geographically associated risk factors such as EHE characteristics, demographics, health status, and household cooling characteristics interact to produce HRIs and related outcomes. Critically, the model built here assumes that EHE frequency, duration, and intensity are independent of one another, and there are studies that suggest that, historically, this albeit simplifying assumption is not entirely unreasonable (Habeeb et al., 2015; Oswald & Rood, 2014). That said, we presume heat waves will generally become more frequent, intense, and longer in duration (Domeisen et al., 2023; Lyon et al., 2019). In fact, studies are beginning to demonstrate an association between these characteristics which may in fact be getting stronger with global warming (Mazdiyasni et al., 2019). Similarly, our model assumes that the 18 corresponding daily HRI risk functions do not vary across regions, but there are in fact numerous studies suggesting important regional differences in HRI risk, given the same EHE exposure, even when controlling for pre‐existing conditions (Vaidyanathan et al., 2019). Explanations for such differences vary, but adaptation is one plausible factor: people living in hotter areas tend to become accustomed, both physiologically and behaviorally, to extreme heat which provides them with a certain degree of resilience (Lee & Dessler, 2023; Sarofim et al., 2016). Another limitation is that for each region, the model multiplied the general prevalence of household cooling, age, and health, where health was itself conditioned on age, to arrive at a joint probability for each of the 18 strata. Thus, the model treated, within each region, household cooling behavior as independent of health status and by extension age. Our model too made the simplifying assumption that the 18 distinct exposure‐response functions adhered to the same basic sigmoidal shape. Furthermore, calibration of our daily HRI risk functions linking HI, age, health, and household cooling to daily HRI probabilities was limited to fitting predicted morbidities, by age and race, to the results of the one CONUS study (Knowlton et al., 2009) of an historical EHE, the 2006 California heat wave, for which heat‐attributable morbidities by age and race were available. Beyond this one study, no data were available to test calibrations across the 18 functions which relied instead on gross estimates of relative risk within each of the three separate stratifications (age, health, and household cooling). As discussed below, these assumptions can and should be tested and refined against place‐ and time‐specific data on EHEs and appropriately stratified HRIs.

As stated, our model estimated morbidity by race using regional conditional probabilities based on 2020 RECS data associating race with household cooling and whether or not the household experienced an HRI at any time during the past year. While we attempted to adjust for changing race distributions over time, these adjustments were based on national Census projections. Furthermore, regional conditional probabilities of race affiliation that were estimated for the duration of the simulation time frame (2005–2040) were based on a calibration that was itself fixed to the 2020 RECS measurements. Similarly, and following other model projections of heat and HRI, we assumed a fixed relationship between surface temperature readings and actual temperature exposure (Sarofim et al., 2016). Future extensions of this model would test the sensitivity of the results to changes in these important parameters. Estimates of population exposures to EHEs would be improved by modeling EHE characteristics at more granular levels of resolution by for example, adding an MSA dimension to the model array. Finally, our model considered only the 53 largest MSAs in CONUS. The model's HRI and cost projections therefore probably represent conservative estimates of HRIs for CONUS urban, peri‐urban, and suburban areas taken as a whole.

A fourth objective of this study was to define a starting point for extending and refining the general model at more local scales that may inform planning to address different community vulnerabilities to extreme heat and HRIs. The model would be improved with analysis of EHEs and their characteristics and associated hospital data for specific localities and periods which could then be used to derive multivariate logistic regression coefficients that predict daily HRI probabilities by relevant strata. Longitudinal studies of localities would furthermore help calibrate exposure‐response functions with respect to deviation in EHE characteristics from historical norms, providing more accurate assessments of HRI risks for communities. When investing in heat risk mitigation, local planners and decision‐makers must consider where, how, and even when such investments should be made that will address a complex array of technical, fiscal, political, and community objectives (Makido et al., 2019). Appropriately calibrated daily HRI risk functions for relevant groups would help to identify investments that are more likely to generate the largest reductions in HRIs and costs in an equitable manner. Adding an additional layer to the model centered on interventions introduces new challenges but also opportunities to not only specify but also calibrate the critical links in particular contexts. Pivotal to such modeling will be estimating “treatment‐response” functions that contemplate counterfactual scenarios. But here too empirical studies like Wang et al. (2024)'s study of the nonlinear scaling effects on the cooling efficiency of tree canopy cover, can be leveraged in estimating such functions. Beyond this, it is important to recognize that extreme heat impacts individuals differently for a variety of reasons and to develop risk‐ameliorating approaches that are population‐specific; develop public health protocols that communicate the effects of heat illness; and offer communication plans that reach those who are otherwise “hard to engage.”

## 5 Conclusions

The extent to which studies can provide accurate predictions of future HRIs will largely depend on the robustness, accuracy, and integration of relevant data sets. Such models will need to consider physical geographies, demographics, and behaviors for predicting HRIs. By drawing on earlier studies and data sets that provide the component parts of an exposure‐response model, we developed an SD model that integrates data to operationalize interactions of climate, geography, demographics, and household cooling behavior.

The future of such models will include systematic tests of the effectiveness of heat risk‐mitigating strategies. Many studies are beginning to demonstrate the importance of, for example, increasing albedo or shade canopies. However, we still know relatively little about how risk factors such as geography and behavior interact which would inform mitigations that target specific communities. One distinct advantage of SD models is the ability to continue refining risk assessment models as new studies and data sets are published, while also appending risk mitigation modules to evaluate heat risk‐mitigating interventions. The current model provides a baseline for such developments, particularly at more local scales where exposure‐response functions and thresholds for action can be calibrated to historical data. Given the ever‐increasing incidence of heat‐related morbidities and mortalities, we need to use every tool available to us to avert an otherwise largely preventable public health emergency.

## Conflict of Interest

The authors declare no conflicts of interest relevant to this study.

## Supporting information

Supporting Information S1

## Data Availability

Additional information related to stratified daily HRI probability bounds is provided in Supporting Information S1. NEMAC projections of major MSAs used to interpolate daily mean number of EHEs are available at https://crt‐climate‐explorer.nemac.org/. For each MSA (see Table 1), users should select the county corresponding to it and navigate to its Climate Graphs page. Users should select the temperature threshold. For this study, except for the Northwest region, we used a daily minimum temperature (T min) threshold greater than 80 degrees F (26.67°C). For the Northwest region, we used a daily maximum (T max) greater than 100 degrees F (37.78°C) threshold. Annual projections of days per year meeting the threshold can then be downloaded. National US Census population projections by race are available at https://www.census.gov/data/tables/2023/demo/popproj/2023‐summary‐tables.html (U.S. Census Bureau, 2023). Users should select Table 5. US regional population projections by age are available at https://view.officeapps.live.com/op/view.aspx?src=https%3A%2F%2Fwww.coopercenter.org%2Fsites%2Fdefault%2Ffiles%2F2025‐01%2FNationalProjections_ProjectedAgeSexDistribution_2030‐2050.xlsx&wdOrigin=BROWSELINK (Weldon Cooper Center for Public Service, 2024). Estimates for health status as a function of age were taken from the National Health Center for Statistics Data Query System which houses data from the National Health Interview (C.D.C., 2026). Researchers can access the data on health status prevalence by age group at https://www.cdc.gov/nchs/dqs/index.html and selecting the following Topic filters: Fair or poor health status in children and Fair or poor health status in adults. For each filter, selecting Age Group in the Group filter will provide prevalence by age group. The time period used for this study ran from 2019 to 2022. The 2020 RECS data used to estimate distribution of household cooling statuses and to make race attributions of HRIs are available at https://www.eia.gov/consumption/residential/ (U.S. Department of Energy, 2025). Researchers can access the master data file and codebook at https://www.eia.gov/consumption/residential/data/2020/index.php?view=microdata. The SD model was built using Stella® software (https://exchange.iseesystems.com/.) The engine runs on integration of ordinary differential (“flow”) equations (ODEs). The model code is available at the Zenodo Repository (Brown, 2025). Rights are licensed under the Creative Commons Attribution 4.0 International.
