# Supplementary material for: Predicting Urban Heat‐Related Illness Across U.S. Climate Regions and Demographics
Source: Geohealth. 2026 Jun 9;10(6):e2025GH001655. doi: 10.1029/2025GH001655 (PMC13248521; doi:10.1029/2025GH001655)
Supplement: Supplementary file 1 — Supporting Information S1 [file GH2-10-e2025GH001655-s001.pdf]

GeoHealth

Supporting Information for

Predicting Urban Heat-Related Illness Across U.S. Climate Regions and Demographics

Stephan E. Brown[1] and Vivek Shandas[2]

[1]CAPA Strategies

[2]Department of Geography, Portland State University

Corresponding author: Stephan Brown (sb@capastrategies.com)

Contents of this file

Text S1
Table S1

Introduction

Following explains the process that was used to derive the daily HRI probabilities for each of the 18 demographic groups at the low and high heat index (HI) extremes. This explanation is followed by a table which presents the final distribution of daily HRI probabilities at the extremes along with the risk ratios (RR) within each stratum, using a defined reference group and holding the other two strata constant.

It is noted that the RRs range from 0.59 to 10.80. It is furthermore noted that RRs do not strictly adhere to an ordinal progression of risk within each stratum of categories, and that there is a systematic positive-risk bias in the 0-4 age group compared to the 65+ age group.

Text S1.

The model estimated the daily probability of experiencing HRI at a given HI value for 18 groups stratified by age, health status, and household cooling. Although all 18 of these exposure-response functions were sigmoidal in shape, the lower and upper values of each EHE exposure-HRI response function were estimated to satisfy multiple conditions. First and most generally, the functions as a set were evaluated by how well the projected nationally aggregated HRI estimates matched national estimates from other studies (McGeehan & Mirabelli, 2001; Woolf et al., 2023). A second condition was to arrive, through multiple iterations, at reasonable ratios of risk both within each group across the HI range of 100- and 130-degrees F (37.78-54.44 ℃) as well as within each of the three general strata at both extremes, comparing to a reference category while

holding the other strata constant. The reference categories for age, health, and cooling were: 5-64, Good Health, and AC, respectively. The model settled on a uniform (regardless of stratification) 100 multiplier when comparing HRI risk at 100 degrees F (37.78 ℃) versus 130 degrees F (54.44 ℃). That is, if a person has, on a given day with an outside temperature of 100 degrees F (37.78 ℃), a 1/10,000 chance of experiencing an HRI, the same person has a 1% chance of experiencing an HRI when the outside temperature is 130 degrees F (54.44 ℃). Risk ratios (RR) within strata, holding the other two strata constant, ranged from 0.59 to 10.80. The RRs do not strictly progress "ordinally" from the reference group. This is perhaps most clearly seen when comparing the daily HRI risk of adults relying on fans to their AC-using counterparts. Also evident is a systematic positive risk-bias in the 0-4 age group compared to the 65+ age group.

Table S1 presents the results of the final distribution of minimal and maximal daily HRI rates which fed the model. These parameters were furthermore tested by simulating the HI-days of the 2006 California HW and comparing the model's HRIs projections by age for the West U.S. Climate Region to Knowlton et al. (2009)'s study of that HW.

Table S1. Estimated daily HRI risk ratios by household cooling, age, and health

AC status	Age	Health	Estimated daily HRI probability at 100 degrees F heat index	Estimated daily HRI probability at 130 degrees F heat index	RR over heat index range	RR over Health range (Good Health = 1, *ceterus paribus*)	RR over AC range (AC = 1, *ceterus paribus*)	RR for Age (5-64= 1, *ceterus paribus*)
No AC or fan	0-4	Good Health	0.00038	0.038	100		4.75	4.22
No AC or fan	0-4	Poor Health	0.00101	0.101	100	2.66	3.88	4.04
No AC or fan	5-64	Good Health	0.00009	0.009	100		3.00	
No AC or fan	5-64	Poor Health	0.00025	0.025	100	2.78	2.27	
No AC or fan	65+	Good Health	0.00011	0.011	100		3.67	1.22
No AC or fan	65+	Poor Health	0.00038	0.038	100	3.45	2.71	1.52
Fan only	0-4	Good Health	0.00019	0.019	100		2.38	10.80
Fan only	0-4	Poor Health	0.0005	0.05	100	2.63	1.92	6.25
Fan only	5-64	Good Health	0.0000176	0.00176	100		0.59	
Fan only	5-64	Poor Health	0.00008	0.008	100	4.55	0.73	
Fan only	65+	Good Health	0.00002	0.002	100		0.67	1.14
Fan only	65+	Poor Health	0.00015	0.015	100	7.50	1.07	1.88
AC	0-4	Good Health	0.00008	0.008	100			2.67
AC	0-4	Poor Health	0.00026	0.026	100	3.25		2.36
AC	5-64	Good Health	0.00003	0.003	100			
AC	5-64	Poor Health	0.00011	0.011	100	3.67		
AC	65+	Good Health	0.00003	0.003	100			1.00
AC	65+	Poor Health	0.00014	0.014	100	4.67		1.27

References

Knowlton, K., Rotkin-Ellman, M., King, G., Margolis, H. G., Smith, D., Solomon, G., Trent, R., & English, P. (2009). The 2006 California heat wave: impacts on hospitalizations and emergency department visits. *Environ Health Perspect*, *117*(1), 61–67. https://doi.org/10.1289/ehp.11594

McGeehan, M. A., & Mirabelli, M. (2001). The potential impacts of climate variability and change on temperature-related morbidity and mortality in the United States. *Environmental Health Perspectives*, *109*, 185–189.

Woolf, S., Morina, J., French, E., Funk, A., Sabo, R., Fong, S., Hoffman, J., Chapman, D., & Krist, A. (2023). *The health care costs of extreme heat* (Center for American Progress Report, Issue. C. f. A. Progress. https://www.americanprogress.org/article/the-health-care-costs-of-extreme-heat/
